# 320 Slice CT in Imaging of Congenital Heart Diseases in Infants: A Single-Center Experience

**DOI:** 10.7759/cureus.13348

**Published:** 2021-02-15

**Authors:** Gayathri Sreedher, David Bruckman, Shankar Srinivas Ganapathy

**Affiliations:** 1 Pediatric Radiology, Akron Children's Hospital, Akron, USA; 2 Statistics, Biostatistical Solutions, LLC, Cleveland, USA

**Keywords:** infant cardiac imaging

## Abstract

Objective

The study was conducted to evaluate the best possible imaging technique for neonatal cardiac imaging including optimal injection techniques, intravenous line placement, expected radiation dose, and need for sedation while performing the study on a 320 slice Toshiba® Aquilion ONE® scanner. Study results can be used to optimize imaging parameters for maximum clinical yield. We provide representative images of our cases.

Methodology

Cardiac CTs performed on infants less than one year of age at the time of study were evaluated. Data collection included radiation dose, duration of the scan, heart rate, type and route of contrast injection, need for sedation or general anesthesia and quality of study including image contrast and motion artifacts.

Results

Average age of infants at the time of scan was approximately two months. Prospectively gated volumetric scans performed within one heartbeat with a single gantry turn formed the majority of studies. Average effective dose was below 1 mSv. Several patients were scanned without any sedation. Most studies were deemed diagnostic and of superior quality on a 4-point scale. Qualitative image analysis revealed an excellent intraclass correlation between two raters.

Conclusion

Parameters needed for successfully performing cardiac CTs with a high degree of diagnostic quality in neonates were identified. For infants below a year hand injection of Isovue 300 in a 24 G peripheral upper extremity IV line with real-time contrast bolus monitoring and manual start to scanning is adequate when being scanned on a 320 slice Volumetric scanner with prospective auto-target EKG gating. Sedation may not be necessary for infants when wrap and feed techniques and free breathing are employed. Radiation doses utilizing this technique were uniformly low.

## Introduction

Congenital heart disease (CHD) is a leading cause of cardiac morbidity and mortality in the less than one-year-old age group. It is estimated that between 2.5 to 3 infants/1,000 live births have severe CHD needing advanced cardiac care [1]. Fetal echocardiography has a high specificity for detecting major malformations and guide the post-natal course of treatment [2]. Post-natal echocardiography and catheter angiography are the mainstay of imaging in congenital heart diseases. However, CT is increasingly being utilized in lieu of diagnostic catheter angiography to better define the vascular anatomy. CT is mainly utilized to evaluate the extracardiac vessels as an adjunct to echocardiography [3]. With the advent of fast volumetric scanners coupled with techniques to significantly reduce radiation dose, infant cardiac CT has become popular in our institution. Here we present a review of factors associated with effective cardiac CT scans in children less than a year of age performed over a three-year period. In addition, we test the reliability of a 4-point scale to evaluate image quality using the 320 slice Toshiba® Aquilion ONE® scanner.

## Materials and methods

**Figure 1:**
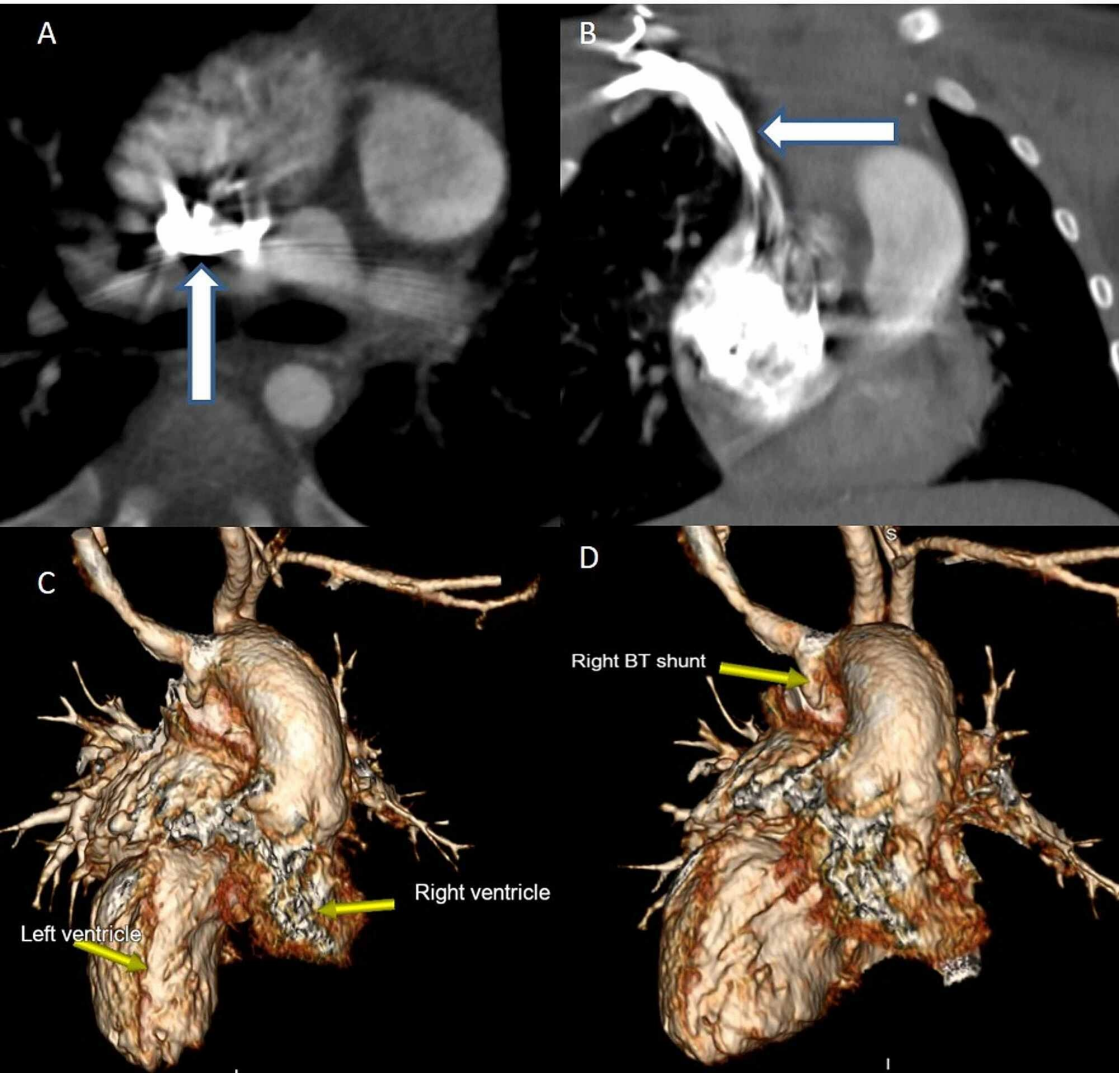
A and B: Axial and coronal images show streak artifacts (arrows) of moderate severity in the SVC in a patient with L-TGA (Congenitally corrected). C: 3D recons depict the great vessel origins. D: 3D recon depicts the B-T shunt.

Following Institutional Review Board approval, all cardiac CTs performed on infants (less than one year of age at the time of study) in our department from November 2014 to December 2017 were evaluated. A total of 40 studies were performed (38 patients). Data were collected on radiation dose, duration of scan, heart rate at the time of study, type of contrast and route of injection, need for sedation or general anesthesia (GA), and quality of study including motion and streak artifacts if any and quality of 3D reconstruction (Figure 1).

All CTs were performed on a 320 multislice Toshiba Medical Systems Aquilion ONE ® scanner with free breathing. Manual scan trigger was used to visually monitor contrast passage in the region of interest. All patients received weight appropriate dose of Isovue 300 intravenous iso-osmolar contrast. 

Radiation dose was recorded as CT Dose Index Volume (CTDI vol) and dose length product (DLP) from the scanner data. Effective dose per DLP was calculated using the conversion factors for pediatric patients. We employed the coefficient proposed by the American Association of Physicists in Medicine (AAPM) working group for calculating normalized effective dose per DLP for pediatric patients over the chest on CT. The coefficient used for the 0-year-old patient/neonate’s chest was 0.039 mSv mGy^-1^ cm^-1^ [4]. 

Images were post-processed on Vitrea® (TR: Vital Imaging) workstation to generate 3D surface shaded reformats. Diagnostic quality of the study on conventional cross-sectional images was rated by two raters (GS and SG) on a 4-point scale: 1 - poor - significant artifact or noise prevents evaluation of vessels and their distinction, 2 - fair - can see major structures but cannot determine any useful information on their state such as appropriate measurements, smaller connections, etc., 3 - good - can separately see all major vessels, their branches/connections with at most mild motion degradation or noise, 4 - excellent - all vessels and structures seen separately with excellent contrast opacification of the vessels of interest. 

To evaluate signal-to-noise ratio (SNR) and contrast to noise ratio (CNR), approximately 5 mm^2^ region of interest was drawn at the ascending aorta and main pulmonary artery level. Density in terms of Hounsfield units (HU) was quantified as signal. Average noise in the region of interest (ROI) was recorded. Quantitative evaluation of the images was done with contrast noise ratio between the aorta (or pulmonary artery) and muscle, calculated as signal in aorta (or PA) - signal in paraspinal muscle divided by average noise (average of noise between aorta, PA and muscle). SNR at both the ascending aorta and pulmonary artery was obtained. 

Interrater and intrarater reliability testing agreement on ordinal scaled responses was measured. intraclass correlation coefficients [5], ICC and confidence intervals were reported. Assuming that these raters possess similar training and characteristics to others who could be involved in this reliability study, we considered the ICC as a two-way random effects model with single raters evaluating the same set of target images for absolute agreement on our proposed scale. Based on this, we assumed an ICC (2,1) form as promoted by Koo and Li [6]. General linear modeling under quasi-independence is preferred over a weighted kappa analysis [7]. SAS Enterprise Guide version 9.4 was used for all analyses (SAS Institute, Cary, NC).

## Results

Average age of the infants at the time of the CT was 61.35 days, approximately two months (range: 1-343 days of age). Three children were above 200 days of age. Thirty-nine of the 40 studies were performed as volumetric acquisition with prospective gating. Thirty-four studies were completed in one heartbeat with a single turn of the gantry. Four studies required imaging over two heartbeats and one study was completed in three heartbeats. Each gantry turn was 0.35 seconds on the Toshiba Aquilion®. One study was done as retrospectively gated helical study over 10 heartbeats. Average RR interval was 403.16 ms (range: 288-585 ms) translating to an average heart rate of 148 beats per minute (60,000/RR). Three patients had additional delayed venous phase scan done to evaluate the venous anatomy better. The three cases included a tetralogy of Fallot status post unifocalization of aortopulmonary collaterals, a case of heterotaxy where systemic venous return needed to be assessed and a third case of pulmonary hypoplasia where a delayed scan to assess pulmonary veins was done. Of note, we did not evaluate the utility of such delayed scans in diagnosis.

All but one study was acquired at 80kV. The average radiation dose in terms of dose length product was 21.91 mGy*cm. This is the average of the total DLP acquired over the scan including smart prep steps. This translates to an average effective dose of 0.855 mSv (σ = 0.49mSv) using the 0.039 mSv mGy^-1^ cm^-1^, coefficient proposed by the AAPM working group [4]. The range of effective dose was 0.36 mSv to 1.53 mSv for single run prospective CTs. The range of effective dose was0.86-2.27 mSv when a second venous phase was acquired with prospective gating (three patients) and effective dose was 2.89 mSv for our single retrospectively gated CT. 

**Figure 2:**
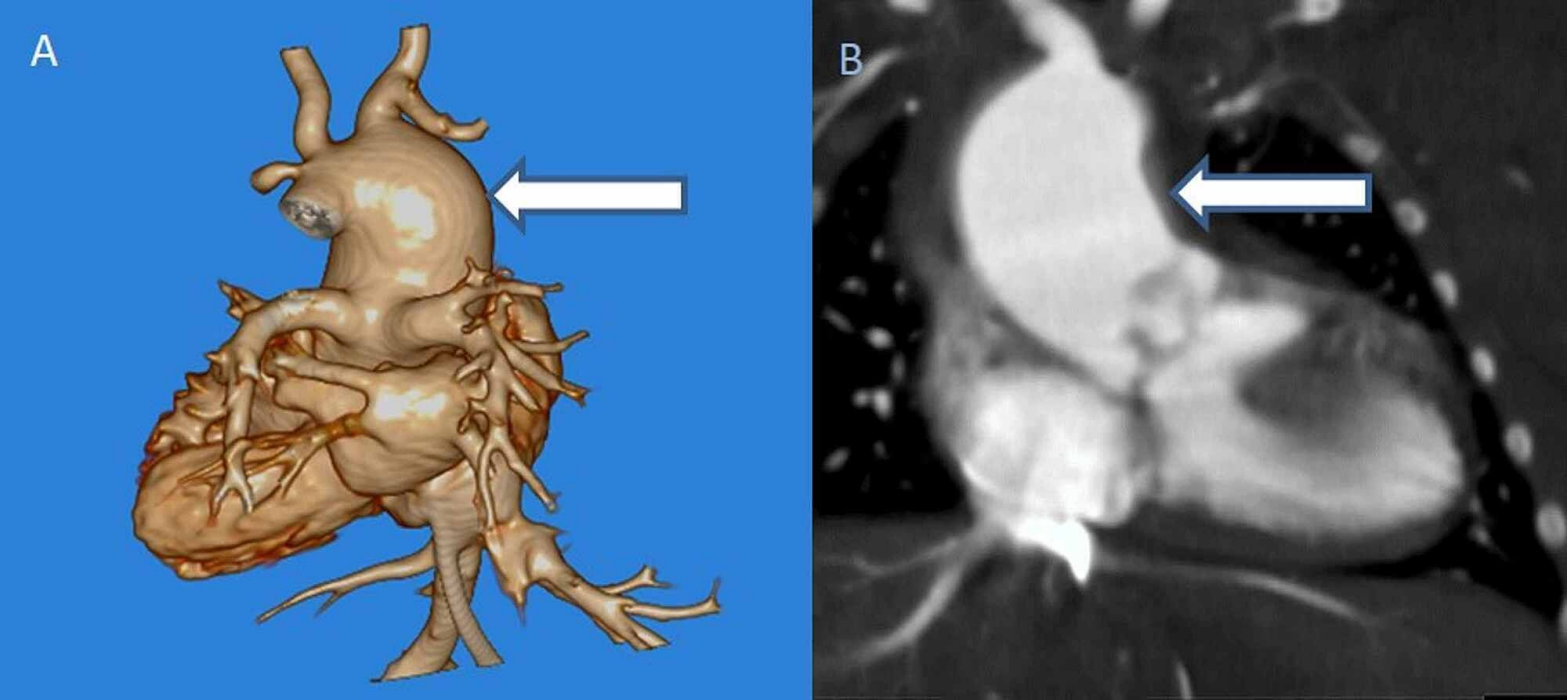
A (3D) and B (coronal) images depict Type 2 truncus arteriosus (arrows). Injection was through the UVC in this one-day-old. UVC: umbilical venous catheters.

Of the 39 studies for whom mode of injection was available, 37 infants had a hand injection and two had a power injection. Both power injections were at 2.1 mL/sec flow rate. Average dose of contrast injected was 9.15 mL. All patients had Isovue 300 as IV contrast agent. Thirteen patients had a 24 G IV placed for the study. Four patients had a 22 G IV. Three patients had a peripherally inserted central catheter (PICC) line and one had a femoral line. Four patients had injections through the umbilical venous catheters (UVC) (Figure 2).

Seventeen patients were documented to have upper extremity IV lines most of which were in the antecubital vein by our hospital convention. Data were not available on the route of injection on the remaining three patients. Of all the patients, 28 had an upper extremity injection with an IV line in either the hand or the antecubital vein region. Fourteen of these were on the right side while an equal number were on the left upper extremity. These findings are tabulated in Table 1. 

**Table 1:** Contrast administration of Isovue 300. PICC: peripherally inserted central catheter.

Characteristic	N	Percent
Total studies	39 (data not available for one)	100%
Hand injection	37	94.90%
Power injection - 2.1mL/sec	2	5.10%
Injection types for known cases		
24 G IV	13	33.30%
22 G IV	4	10.30%
Upper extremity injections (hand and antecubital vein)	28	70.00%
PICC line	3	7.70%
Femoral line	1	2.60%
Umbilical line	4	10.25%
Unknown injection site	3	7.70%

Fifteen patients had no sedation for the procedure. Eleven patients came from pediatric intensive care unit (PICU) and presumably were either intubated or sedated by the PICU team for the study. Fifteen patients were admitted to the neonatal intensive care unit (NICU) at the time of the study. A wide range of pathologies were encountered (Table 2).

**Table 2:** Range of pathologies (major anomaly listed as most patients had several associated anomalies).

Major pathology	N
Hypoplastic aortic arch, coarctation of aorta and post coarctation repair	8
Transposition of great arteries (D TGA and Congenitally corrected L TGA)	3
Double outlet right ventricle	2
Truncus arteriosus	4
Total anomalous pulmonary venous return	4
Partial anomalous pulmonary venous return	2
Double outlet left ventricle	1
Tetralogy of Fallot, pulmonary atresia and major aortopulmonary collaterals	8
Aberrant aortic arch branching/aberrant subclavian	3
Miscellaneous (ring and sling, dextroversion, congenital diaphragmatic hernia and William syndrome)	5

**Figure 3:**
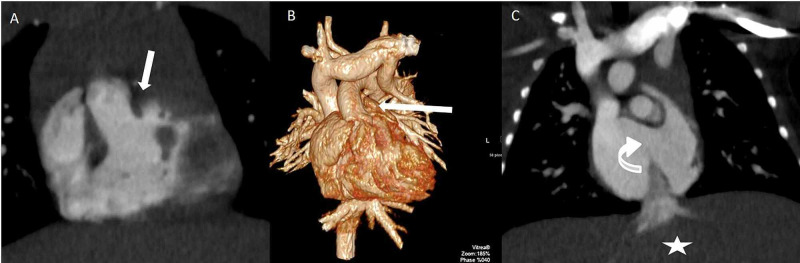
Coronal (A and C) and 3D (B) images in a one-day-old with complex CHD – heterotaxy with midline liver (starred), total anomalous pulmonary venous return (TAPVR) (not shown), DORV with side-by-side origin of great vessels (straight arrow) and ASD (curved arrow) are all well depicted. CHD: congenital heart disease; DORV: double outlet right ventricle.

**Figure 4:**
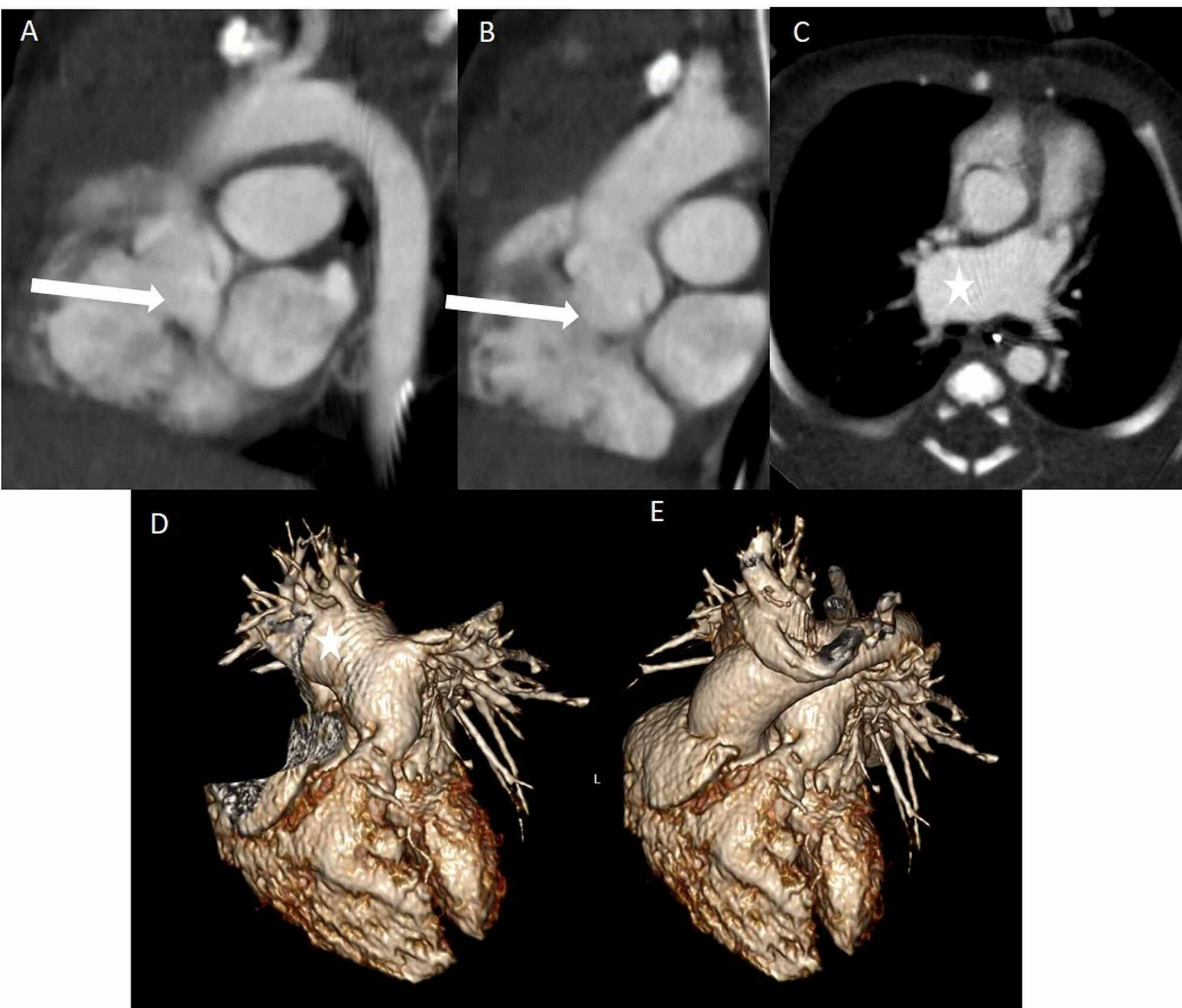
A and B - sagittal images in a one-day-old with TOF (with absent pulmonary valve) show the aorta overriding the VSD (arrows) and C - axial images show dilated RPA (star) compressing the main stem bronchus, also seen on 3D recons (D and E). TOF: Tetralogy of Fallot; VSD: ventricular septal defect; RPA: right pulmonary artery.

Most patients had complex CHD with several coexisting pathologies (Figures 3, 4).

 

Perfect agreement on the four-level qualitative scale was achieved in 92.5% of our sample. Qualitative image quality analysis revealed an intraclass correlation coefficient (ICC (2,1)) of 0.79 reflecting good reliability of the ICC estimate [6]. None of the studies was classified by either rater as 1 on the 4-point scale (1 - poor - significant artifact or noise prevents evaluation of vessels and their distinction). Five studies were rated as 2 by at least one rater (2 - fair - can see major structures but cannot determine any useful information on their state such as appropriate measurements, smaller connections etc.). The remaining studies were rated either 3 - good or 4 - excellent by both raters. 

Quantitative image quality analysis revealed average SNR of 27.69 at ascending aorta, ranging from 7.79 to 87.92. SNR at the main pulmonary artery averaged at 25.17, ranging from 8.43 to 87.92. Mean noise in the images averaged at 21.79. Using the best contrast to noise ratio between aorta and PA, the average CNR was 25.97, ranging from 8.2 to 82.48. 

Coronary artery visualization is one of the markers of cardiac CT quality [8] and clinically significant motion artifact. In 19 cases (47%), we were able to see both coronary arteries and in 14 cases at least one was seen. 

## Discussion

In the last couple years, there have been relatively few studies evaluating the feasibility and successful acquisition of infant cardiac CT’s on the newer 320 slice CT scanners. Cardiac imaging on the Toshiba Acquilion ® comes with several tools to improve imaging quality and decrease radiation including a pediatric volumetric chest/cardiac imaging mode that includes volumetric acquisition, target mode, prospective EKG gating, iterative reconstruction and half scan reconstruction. The study by Jadhav et. al suggests that target mode prospective ECG-gated studies yielded images of superior quality compared to the ungated helical CT [8]. 

Our high inter-observer agreement using intraclass correlation on the study quality with more than two-thirds of the ratings in the highest category of excellent study quality are promising and a testament to the success of the protocols used. While a more elaborate system of image analysis was used by Jadhav et al [8], the results were similar to ours with high kappa ratings (0.85) and excellent image quality ratings in target mode cardiac CTs. 

Similar to the study by Saake et al [9], we found that image quality was good with manual injection. We did not separately study the added effect of power injection due to small number of power injections (n = 2), nor did we dilute our contrast with saline. The CNR (averaging at 25.97 in our study) was slightly superior to their study although we had a wide range of CNR. 

Background radiation exposure from natural sources is about 3mSv per year in the United States. In a multi-center multi-vendor study demonstrating variations in radiation dose metric of pediatric cardiac CT in Asia, Hui et al [10] found doses ranging from 1.5 to 3.2 mSv which is similar to our doses that ranged from 0.36 to 2.89 mSv. A study by Gao et al found even lower radiation doses with an average of 0.53+/-0.15 mSv [11]. Using our data from single runs and excluding the patients who had a venous phase or retrospective gating our radiation doses would be comparable at 0.36-1.53 and lower than those of Hui et al. We also used a higher coefficient ratio for radiation dose calculation than Gao et al in patients above four months of age. We used the same coefficient regardless of age of infant, for the sake of convenience and uniformity. Doses reported by Saake et al [9] also are similar with mean doses of 0.8+/-0.6mSv. Geryes et al [12], Han et al [13] and Al-Mousily et al [14] reported similar doses. However, note must be made that these doses are a mere approximation as they all vary slightly depending on the conversion factor, body phantom size chosen as well as the study kVp. Furthermore, the doses are similar to a phantom study done by Lee et al [15]. 

Mean heart rate in our group was 153 bpm. The center of data acquisition was set at 40% of the R-R interval automatically by the scanner given the high heart rates. This heart rate is comparable to the mean heart rate of 129.19+/-14.52 bpm in the study by Gao et al [11]. Similar to prior studies our patients also underwent a low kVp study with all but one study performed with a tube voltage of 80 kVp. The use of low tube voltage in addition to the low tube current translates to a lower radiation dose. The lower tube voltage also helps with an increase in contrast to noise ratio when iodinated CT contrast is used. Our CNR averaging at 25.97 is similar to their study with CNR of 28.19+/-13. Our CNR is only slightly lower than the study by Jhadhav et al [8]. Although both studies had a slightly different internal control for the calculation of contrast than our study. Our CNR was better than those reported by Saake at al [9]. 

Of interest is the prevalence of non-sedated (free breathing infant) studies in our study. We only had 15 patients who either had sedation or were intubated. At least 11 patients had no sedation. Data for several was missing and although they had no endotracheal tubes it wasn’t documented as to whether they received any sedating medicines. The adequacy of free breathing non-sedated cardiac CT’s in infants has been previously established [8,11]. 

Except for three cases, where it was deemed necessary to obtain a delayed phase imaging, all patients had single-phase imaging. This is in tandem with other infant cardiac CT studies where only a single-phase imaging is performed using single first-pass contrast enhancement. 

Coronary artery visualization at 47% for both arteries was lower than expected. The small size of patients, motion artifacts from high heart rate and lack of surrounding fat for adequate delineation were felt to be the culprits. The visualization is certainly less than what is quoted in literature (Goo et al [16] with 79%-89%). Coronary visualization may also have been affected by the absence of power injection on most of our cases. Power injection may improve contrast to noise ratio, thereby improving the conspicuity of coronary arteries. 

Our study however suffers from several limitations. The most important is the lack of complete data regarding the patient’s sedation versus wrap and feed scanning status. This limits our ability to recommend wrap and feed technique as the first-line strategy for infant cardiac CT imaging. Certainly, it wouldn’t work for the older infants who would move significantly despite swaddling/restraints. In the discussion of radiation doses between different studies, there is inconsistency in the coefficient utilized to calculate effective dose data. This should be kept in mind while comparing them across different studies. Incomplete data regarding heart rate was another limitation however given the uniformly higher heart rates in the 30/40 patients that had the data, it is reasonable to assume that all patients had a high heart rate as is expected for this age group. Finally, we did not correlate with echocardiography or surgical findings. It is assumed that the adjunctive role of cardiac CT in optimal management of complex congenital heart disease is well established and proving the supplemental nature of CT was not our intention.

## Conclusions

Parameters needed for successfully performing cardiac CTs with a high degree of diagnostic quality in neonates were identified. For infants below a year hand injection of Isovue 300 in a 24 G peripheral upper extremity IV line with real-time contrast bolus monitoring and manual start to scanning is adequate when being scanned on a 320 slice Volumetric scanner with prospective auto-target EKG gating. Sedation may not be necessary for infants when wrap and feed techniques and free breathing are employed. Radiation doses utilizing this technique were uniformly low and sub millisievert.
